# Bone quality assessment for total hip arthroplasty with intraoperative trabecular torque measurements

**DOI:** 10.1186/s13018-014-0109-0

**Published:** 2014-11-13

**Authors:** Matthias CM Klotz, Nicholas A Beckmann, Rudi G Bitsch, Elisabeth Seebach, Tobias Reiner, Sebastian Jäger

**Affiliations:** Laboratory of Biomechanics and Implant Research, Center for Orthopedics, Trauma Surgery and Spinal Cord Injury, Heidelberg University Hospital, Schlierbacher Landstraße 200a, 69118 Heidelberg, Germany

**Keywords:** Total hip arthroplasty, Bone strength, Bone quality, Torque measurement, Biomechanical

## Abstract

**Background:**

In cases of poor bone quality, intraoperative torque measurement might be an alternative to preoperative dual-energy X-ray absorptiometry (DXA) to assess bone quality in total hip arthroplasty (THA).

**Methods:**

Trabecular peak torque measurement was applied in 14 paired fresh frozen human femurs. Here, a 6.5 × 23 mm wingblade was inserted into the proximal femur without harming the lateral cortical bone. Further tests of the proximal femur also evaluated bone strength (DXA, micro-computed tomography (μCT), monoaxial compression test), and the results were compared to the trabecular torque measurement. Student’s *t*-test was used to compare the values of the groups. Pearson product–moment was applied to correlate the values of the peak torque measurement with the bone strength measured by DXA, μCT, and monoaxial compression test.

**Results:**

In the femoral head, the mean trabecular peak torque was 4.38 ± 1.86 Nm. These values showed a strong correlation with the values of the DXA, the μCT, and the biomechanical load test (Pearson’s product–moment: DXA: 0.86, μCT-BMD: 0.80, load test: 0.85). Furthermore, the torque measurement showed a more pronounced correlation with the biomechanical load test compared to the DXA.

**Conclusions:**

The use of this method provides highly diagnostic information about bone quality. Since the approach was adjusted for THA, no harm of the lateral bone stock will result from this measurement during surgery. The results of this initial study employing small sample sizes indicate that this new method is as sensitive as DXA in predicting bone quality and may function as an intraoperative alternative to DXA in THA. Nevertheless, before this method will turn into clinical use, more research and clinical trials are necessary.

## Background

The quality and density of the bone is an important parameter influencing the choice of implant in total hip arthroplasty (THA), because the bone quality of the proximal femur influences the longevity of THA [1]. The use of cemented THA is still important in elderly patients with deficient bone stock and poor bone quality [2,3], but the use of cementless THA and especially bone conserving short stem arthroplasties is increasing worldwide. In osteoporotic bone, the trabeculae of the cancellous bone are deficient and the osteoblastic activity is decreased, which minimizes the ability of bone ingrowth of uncemented stems [1,4-6].

However, in order to assure appropriate fit and primary stability of the implant in osteoporotic bone, the choice of an adequate implant is necessary to guarantee a high survival rate of the prosthesis. It is evident that prior to arthroplasty, the surgeon should have a detailed information about the patient’s bone quality for planning and choosing the optimal implant and determining the postoperative weight bearing.

There are several markers indicating bone structure and bone quality of the patient. In THA, the bone mineral density (BMD) captured by dual-energy X-ray absorptiometry (DXA) is the most frequently used marker to evaluate the quality of bone [7,8]. In addition to the age of the patient, the BMD value is the best clinical parameter to estimate the bone strength. Therefore, the BMD is often considered when deciding which THA implants individual patients may need. However, there are errors, which limit the validity of the BMD captured by DXA. Errors may result from potential sclerosis of surrounding blood vessels, lymph nodes, or muscles. Also, other bone diseases resulting in reduced bone quality such as osteomalacia may be misinterpreted by using DXA [9-12].

A new method to evaluate local bone quality in proximal femur fractures was introduced and reported by the Association for the Study of Internal Fixation (AO Foundation, Davos, Switzerland). In this context, the values of a trabecular resistance torque measurement in the proximal femur using the DensiProbe Hip™ (AO Foundation, Davos, Switzerland) wingblade showed excellent correlations with those of the BMD measured by DXA [11-16]. As the torque measurement was applied in trauma cases, the measurement device was inserted in a retrograde manner or from a lateral distal position into the proximal femur [12,13]. Because the integrity of the lateral cortex is essential for the primary stability in THA and a weakening of the lateral cortical bone enhances the risk for periprosthetic fractures perioperatively, the retrograde approach, which was described by Suhm et al., is not applicable in THA [13].

The purpose of this experimental study was to evaluate an anterograde, i.e., a medial to lateral, transcapital approach into the proximal femur to allow assessment of bone strength using intraoperative trabecular torque measurement in THA. A further aim was to compare and correlate the data of the trabecular peak torque measurement with the BMD as measured using DXA and micro-computed tomography (μCT). Since mechanical properties of trabecular bone are important for implant fixation in arthroplasty [17-20] and reports have indicated a significant correlation between the measurement of BMD by DXA and the measurement of compressive load, a further aim was to compare the trabecular resistance torque in the proximal femur with DXA-BMD, microstructural parameters of μCT, and the maximum monoaxial compression load [21]. Additionally, microstructural trabecular parameters captured by μCT and biomechanical bone strength were also evaluated to prove the validity of the torque measurement. We hypothesized that an intraoperative trabecular peak torque measurement properly estimates bone quality and may function as a surrogate for DXA before THA is carried out.

## Methods

In our experimental study, 14 paired fresh frozen human femurs were used to compare different methods to evaluate bone quality. The mean age of the paired human femurs was 72.0 (45.0–88.0) years. The mean body mass of the donors was 66.9 (37.0–136.0) kg, the mean height was 1.74 (1.57–1.88) m, the calculated average body mass index (BMI) was 21.6 (13.0–38.5) kg/m^2^, and the gender distribution 8/6 (w/m). Donors who had a pathology of the proximal femur in their patient history were excluded. Further exclusion criteria were cancer or myeloproliferative diseases as well as anti-osteoporotic treatment. Despite defining the exclusion criteria, one left proximal femur of a 77-year-old female with no documented hip pathology in the patient history showed a dysplastic femoral head after soft tissue removal. Hence, this femur was excluded from further investigations. One experienced orthopedic surgeon performed all the experiments. This study was approved by the Ethic Committee, University of Heidelberg, Medical School (Ethikkommission der Medizinischen Fakultät Heidelberg, Alte Glockengießerei 11/1, 69115 Heidelberg, Germany).

### BMD measured by DXA

Preoperative standard BMD were measured (QDR-2000 DXA-Densitometer, Hologic Inc., Waltham, MA, USA) at the neck of the proximal femur [22,23]: before the measurement, the densitometer was calibrated using a spine phantom provided by the manufacturer (Hologic Inc., Waltham, MA, USA). The exposure on the femoral neck was carried out in anterior-posterior direction. The bone mineral density was captured in g/cm^2^ and the *T*-score (standard deviation of peak bone mass) was calculated [24].

### Mechanical torque measurement

**Figure 1:**
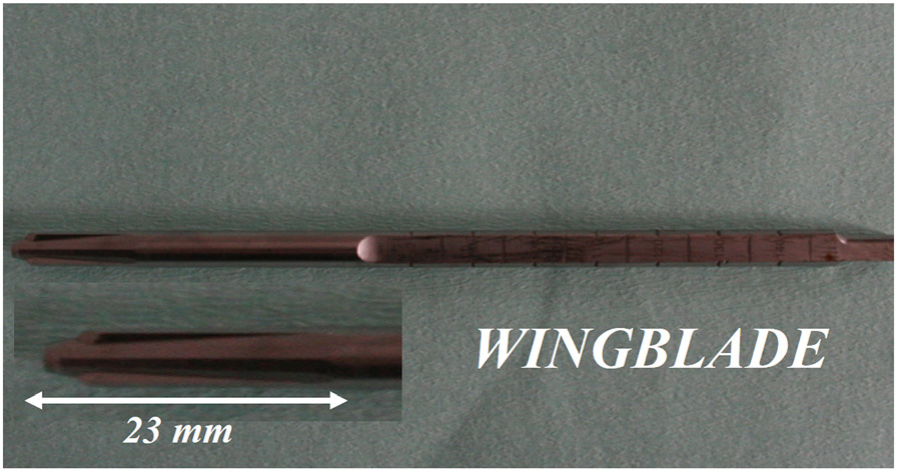
**The wingblade of the torque measurement device (DensiProbe Hip™) had a diameter of 6.5 mm and a length of 23 mm.**

**Figure 2:**
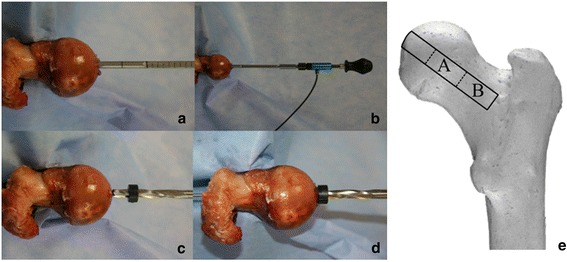
**Trabecular peak torque measurement.** After the insertion of a 2.5-mm K-wire the femoral cortex was opened with a 8.0-mm twist bit to prevent cortical measurement **(c).** A locking device assured a stop at the depth of 15 mm **(d)**. Afterwards, the wingblade was inserted **(a)** and connected to the torque sensor **(b)**, which was then connected to a handle bar for torque measurement. The depth of the first measurement was located in the femoral head at a depth of 15–38 mm **(e: A**). The depth of the second measurement was located in the femoral neck at a depth of 38–61 mm (**e: B**).

Measurement of trabecular peak torque was used to assess the resistance of bone to mechanical failure. The wingblade of the DensiProbe Hip™ (AO Foundation, Davos, Switzerland) was used for torque measurement. The blade was 23 mm in length with a diameter of 6.5 mm (Figures 1 and 2a). The terminal end of the device was connected to a torque measurement probe. We used a torque sensor with a very short axial length optimized for screwing applications. The torque sensor has a range of ±20 Nm, a linearity of <0.02%, an accuracy of 0.1%, and a repeatability of 0.02%. Sensitivity at 20 Nm: 2 mV/V, full scale range 0.1–20 Nm, excitation 2–12 V DC, thrust load limit 580 N, shear load limit 8.6 N (torque sensor D-2431, Lorenz Messtechnik GmbH, Altdorf, Germany) and a handle bar (Figure 2b).

The proximal femur was fixed in a bench vice at the subtrochanteric bone. A 2.5-mm K-wire was drilled into the femoral head along the axis of the femoral neck up to the lateral cortical bone. To guarantee no cortical bone contact, the femoral head was drilled by an 8.0-mm twist bit 15 mm deep (Figure 2c,d).

Afterwards, the peak torque was measured in two different depths (Figure 2e). The first measurement depth (depth one) was located in the femoral head equator with a distance to the medial upper cortex of 15–38 mm (Figure 2a,b,e). The second depth was located in the femoral neck with a distance to the medial upper cortex of 38–61 mm (Figure 2e). A torque measurement device (torque sensor D-2431, Lorenz Messtechnik GmbH, Altdorf, Germany) was connected onto the tip of the wingblade (Figure 2b). The measurements were performed at both depths by rotating the device clockwise around its longitudinal axis until complete breakaway of the trabecular bone was noted. Peak torque was captured using a USB-DAQPad (NI DAQPad-6015, National Instruments Corp., Austin, Texas) and a custom-made real-time measurement software (BoneTorque v1.0).

### Retrieval of bone cylinders

To analyze the trabecular bone in the femoral head by μCT and monoaxial compression tests, bone cylinders with a diameter of 9.0 mm and a length of more than 46 mm were retrieved according to the protocol of Morgan and Keaveny [25,26]: to guarantee a similar bone quality and to avoid damage of the bone cylinders the specimens were retrieved using a coring drill next to the zone of the torque measurements [25]. The corresponding bone area of the torque measurement at depth one was identified and marked. The bone cylinders were kept hydrated, wrapped in plastic, and stored at −20°C in air-tight containers until μCT and compressive load tests.

### Micro-computed tomography

μCT was performed using a Sky-Scan 1076 in vivo X-ray microtomograph (Skyscan, Antwerpen, Belgium). The bone samples were positioned in craniocaudal orientation along the z-axis of the μCT scanner. Depth one (femoral head) was marked on a lateral scout view as the region of interest for scanning. Scanning was performed using a 0.5-mm aluminum filter with the following settings: voxel size 9 μm, voltage 50 kV, current 200 μA, exposure time 1,850 ms, frame averaging 3. Data were recorded every 0.8 degrees of rotation up to 180 degrees. Reconstruction was done using NRecon® software (version 1.6.3.2, Skyscan, Antwerpen, Belgium).

The mean trabecular axis was compared to the main trabecular structural axis in order to screen for misaligned samples, whose off-axis angle was too large. The discrepancy, or off-axis angle, between the samples used and the main trabecular structural axis was less than 20°, and consequently none of the samples were excluded [27].

The following parameters were analyzed with the CT Analyzer software (version 1.10.9.0+, Skyscan, Antwerpen, Belgium): percent bone volume (BV/TV), trabecular number (Tb.N), trabecular thickness (Tb.Th), trabecular separation (Tb.Sp), and μCT-BMD. The specimens were checked for microdamage-induced artifacts according to Nagaraja et al. [28]. To exclude surface and microdamage artifacts, a circular region of interest (ROI) was set in the middle of the bone cylinders with a diameter of 6 mm. The volume of interest (VOI) was determined in its cranial and caudal dimension by starting 500 slices away from the cranial cutting face and taking 501 slices towards the caudal direction which corresponds to 4.5 mm. For the calculation of the microstructural parameters, the lower gray threshold was set to 41 Hounsfield units (HU) and the upper gray threshold was set to 255 HU. μCT-BMD was analyzed as described in the manufacturer’s instructions [29]: a control phantom using water with a gray value corresponding to a HU of zero, and two customized phantoms with defined densities of 0.25 and 0.75 g/cm^3^ of calcium hydroxyapatite were scanned alongside the bone samples. μCT-BMD calibration was performed according to the updated relationship between μCT-BMD, HU, and gray levels [29].

### Biomechanical failure test in monoaxial compression

The bone cylinders were cut distally to a length of 10 mm. Hence, they represented the cranial or medial part of depth one corresponding to measurement in the femoral head. The monoaxial compressive load test was done according to Keaveny et al. [25,26]. Flat platens were attached to each end of the specimen in order to minimize the effects of end artifacts on mechanical testing data [25,26,28]. The resulting pieces with a diameter of 9 mm and a length of 10 mm were loaded by two metal, circular panels compressively, which applied a preload of 1 N for 5 s. Afterwards, the superior panel moved downwards giving a monoaxial compression load with a velocity of 0.1 mm/s.

The maximum compressive strength at failure was measured using a static material testing machine (Zwick GmbH & Co., KG, Ulm, Germany).

### Statistical analysis

An *a priori* power analysis was conducted to adjust the sample size. The data were evaluated descriptively using their arithmetic mean, standard deviation, minimum and maximum. Student’s *t*-test was done to compare the values of the left and right body sides as well as to compare the values of the different peak torque measurements. If Student’s *t*-test was not applicable, a Mann–Whitney rank sum test was applied (*p* <0.05). The Pearson product–moment correlation was used to correlate the data of the BMD measured by DXA and by μCT with those of the torque measurement, the compressive strength and the microstructural trabecular parameters. *Post hoc* calculations using an α of 0.05 were done to calculate the power (1-β) of statistical analysis. For statistical analysis SPSS (SPSS v21, IBM, Armonk, NY, USA) and G*Power (G*Power v3.1.9 for Windows, Heinrich-Heine-Universität, Duesseldorf, Germany) were used.

## Results

### *A priori* power analysis

Using an expected correlation of 0.92 according to previous published results [16], the power analysis (α = 0.05, 1-β = 0.05, tails = two) showed a required sample size of eight specimens to achieve a power of 0.98.

### Bone mineral density

Considering the sample size, there were no significant differences between the left and right body sides with regard to the BMD and *T*-scores (Table 1).

**Table 1:** **The detected and analyzed parameters for each specimen**

**Specimen**	**Body side**	**BMD (DXA)**	**BMD (DXA)**	**Trabecular torque depth 1**	**Trabecular torque depth 2**	**Compressive strength**	**BMD (**μ**CT)**
		***t*** **-value**	**g/cm** ^**2**^	**Nm**	**Nm**	**MPa**	**g/cm** ^**3**^
1	Left	−2.47	0.75	4.19	0.45	5.6	0.22
1	Right	−2.52	0.74	5.43	0.42	4.8	0.24
2	Left	−2.67	0.65	2.18	0.17	1.0	0.23
2	Right	−2.53	0.67	2.21	0.20	2.4	0.24
3	Left	−0.09	1.06	6.86	1.62	5.5	0.47
3	Right	−0.20	1.05	7.01	1.20	8.6	0.50
4	Left	Excluded	Excluded	Excluded	Excluded	Excluded	Excluded
4	Right	−4.71	0.41	2.01	0.13	2.0	0.27
5	Left	−2.64	0.66	2.50	0.14	2.2	0.25
5	Right	−3.04	0.61	3.22	0.10	2.6	0.24
6	Left	−1.80	0.84	4.80	0.55	7.5	0.40
6	Right	−1.98	0.82	4.18	0.68	5.4	0.35
7	Left	−1.95	0.82	5.52	0.79	5.9	0.36
7	Right	−1.86	0.83	6.78	0.82	5.8	0.38
Mean		−2.19	0.76	4.38	0.56	4.6	0.32
Min		−4.71	0.41	2.01	0.10	1.0	0.22
Max		−0.09	1.06	7.01	1.62	7.5	0.50
SD		1.17	0.17	1.86	0.46	2.3	0.10

Values of the torque measurements in the head and the neck (trabecular torque depth 1/2), the bone mineral density (BMD) measured by dual energy absorptiometry (DXA), and micro computed tomography (μCT) and the compressive strength by biomechanical load test. Also shown are the mean value, the standard deviation (SD), as well as the minimum (min) and maximum (max).

### Mechanical torque measurement

The values of the peak torque are also shown in Table 1. Considering the numbers available, there were no significant differences between the left and right body sides concerning the peak torque measurements at depths one (femoral head) and two (femoral neck). At depth two, the values of the peak torque were significantly reduced in comparison to those of depth one (*p* <0.001; 1-β = 0.99).

**Figure 3:**
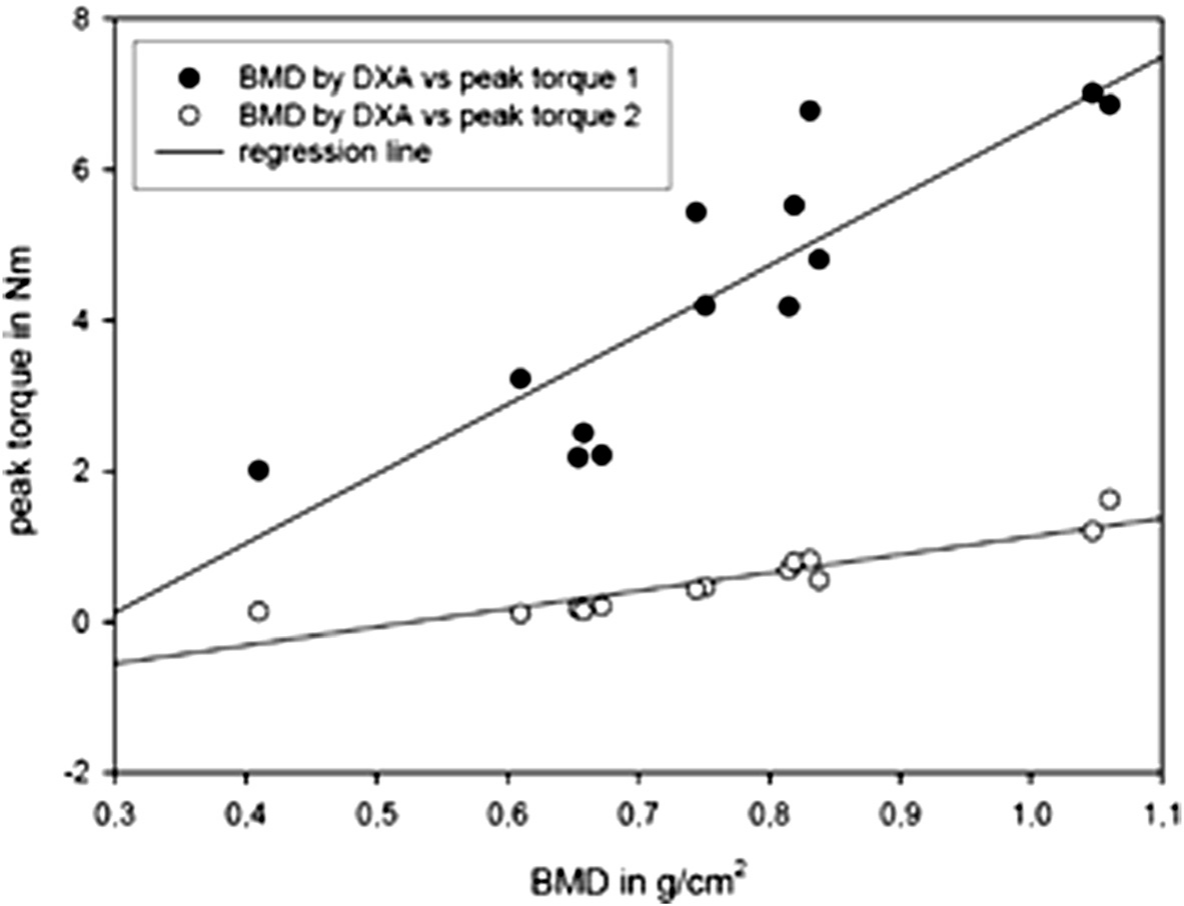
**Correlation analysis (Pearson’s correlation) between the peak torque in the femoral head (peak torque 1) and the femoral neck (peak torque 2) with the bone mineral density (BMD) measured by dual-energy x-ray absorptiometry (DXA).**

There was a positive correlation between the values of the BMD with those of the peak torque at depth one (correlation coefficient 0.86, *p* <0.001; 1-β = 0.99, Figure 3). Furthermore, the values of the *T*-score correlated with the peak torque at depth one (correlation coefficient 0.80, *p* = 0.001; 1-β = 0.99). The values of the peak torque at depth two showed a higher correlation to the BMD (correlation coefficient 0.91, *p* <0.001; 1-β = 0.99, Figure 3) and to the *T*-score (correlation coefficient 0.88,*p* <0.001; 1-β = 0.99) than the values of the peak torque at depth one.

**Table 2:** **The values of the microstructural parameters captured by** μ**CT: percent bone volume (BV/TV), mean trabecular thickness (Tb.Th), mean trabecular number (Tb.N), and mean trabecular separation (Tb.Sp)**

**Parameter**	**BV/TV**	**Tb.Th**	**Tb.N**	**Tb.Sp**	**Compressive strength**
Unit	%	Mm	1/mm	mm	MPa
Value	21.97	0.23	0.95	0.71	0.32
SD	7.4	0.03	0.22	0.13	0.10
Max	35.49	0.29	1.35	0.95	0.50
Min	14.59	0.18	0.65	0.50	0.22

The compressive strength measured by biomechanical load test is imaged. Also shown are the mean values, the standard deviation (SD), as well as the minimum (min) and maximum (max).

### Micro-computed tomography

Considering the limited sample size, the values of the calculated microstructural parameters and the μCT-BMD showed no significant differences between the left and right body sides. The results of μCT data are listed in Tables 1 and 2. There was a positive correlation of μCT-BMD with the BMD measured by DXA (correlation coefficient 0.83, *p* <0.001; 1-β = 0.99). Furthermore, there was a positive correlation between the BMD measured by μCT and the values of the peak torque measurement in the femoral head (correlation coefficient 0.80, *p* <0.001; 1-β = 0.99, Table 3).

**Table 3:** **The correlations (** *p* **<0.05) between the values of the torque measurement, the bone mineral density, the compressive strength, and the other detected and analyzed parameters**

**Parameter**	**BMD (DXA)**	**BMD (**μ**CT)**	**BV/TV**	**Tb.Th**	**Tb.N**	**Tb.Sp**	**Compressive strength**
Torque head	0.86	0.80	0.82	0.63	0.80	−0.77	0.85
BMD (μCT)	0.83	-	1.0	0.85	0.94	−0.91	0.79
BMD (DXA)	-	0.83	0.82	0.82	0.69	−0.67	0.80
Torque neck	0.91	-	-	-	-	-	-

Pearson’s product–moment was used for correlation.

Abbreviations: *BMD* bone mineral density, *DXA* dual-energy X-ray absorptiometry, *μCT* micro-computed tomography, *BV/TV* percent bone volume, *Tb.Th* mean trabecular thickness, *Tb.N* mean trabecular number, *Tb.Sp* mean trabecular separation.

### Biomechanical compressive strength

The values of the maximum compressive strength are listed in Table 1. There were no significant differences between the left and the right body sides due to the limited sample size. There was a positive correlation between the compressive strength and the BMD (correlation coefficient 0.80, *p* = 0.001; 1-β = 0.99). Furthermore, there was a positive correlation between the maximal compressive strength and the peak torque in the femoral head (correlation coefficient 0.85, *p* <0.001; 1-β = 0.99, Table 3).

## Discussion

The purpose of this study was to evaluate the association between bone strength and bone quality as determined by DXA, micro-computed tomography and biomechanical load tests associated with an intraoperative trabecular torque measurement for use in total hip arthroplasty.

A similar method was already shown by Suhm et al. who used a lateral approach in trauma cases [11-13]. Since the integrity of lateral cortical bone in THA is essential to guarantee a press fit anchorage and consequently for primary stability and bony ingrowth, particularly when using short stem arthroplasties, a new transcapital approach was introduced. In this study, the torque measurement was done after dislocating the intact hip without an osteotomy of the proximal femur (Figure 2a,b,c,d). Since many surgeons prefer to perform an osteotomy before dislocating the hip, the torque measurement would only be applicable in the femoral head. In order to enable a torque measurement for both surgical approaches in THA, the peak torque was measured in the femoral head with a distance of 15 mm to the superior cortical bone (Figure 2e). At this location, we found similar peak torque results to those reported in previous studies [11-13]. There was a strong correlation between these trabecular peak torque values and those of the DXA-BMD, which underlines the validity of this measurement. In addition, the values of the trabecular torque measurement showed a significant correlation with the maximum monoaxial compression load. Furthermore, there was also a strong correlation between the trabecular peak torque measurement and the microstructural parameters (trabecular number, trabecular separation, ratio of bone tissue volume) (Table 3), which was measured by μCT and function also as an indicator for bone quality or bone strength. Hence, this shows that the trabecular torque measurement for assessing bone quality and bone strength is consistent with and associated with DXA, because the correlation with the values of the biomechanical compression test and the microtrabecular parameters were similar. Furthermore, monoaxial compression tests provide high sensitivity information about bone strength [30,31]; also, the analysis of the microtrabecular parameters and BMD measured by μCT is more accurate in evaluating bone strength and bone quality than DXA [31-33].

The BMD as quantified by DXA represents data of the femoral neck. Therefore, we decided to carry out the torque measurement values at a second depth corresponding to the femoral neck, even though this area might prove less useful in a clinical setting. At this second depth, the values of the torque measurement showed a stronger correlation with the values of the BMD measured by DXA than did the torque values in the femoral head (Table 3). We did not perform any further investigation at this location. If the measurement process in the femoral neck is improperly carried out, complications may arise which could have an extensive or even detrimental impact on the surgery. For example, if the cortical bone of the femoral neck is injured, fixation of short-stem arthroplasties would be compromised and a larger more distally anchoring prosthesis would be required. Furthermore, measurement in the femoral neck is only applicable before performing the osteotomy of the proximal femur. Given the drawbacks of femoral neck measurement with this device, the peak torque measurement in the femoral head would most likely be more practicable in a clinical setting. Nevertheless, the correlation between the values of the DXA-BMD and the values of the trabecular torque measurement was excellent with a more pronounced correlation in depth two (femoral neck), which had not been not tested by other groups before [11-13].

### Limitations of the study

The study represents the data of a cadaveric study. As this study investigates the practicability of this new method, standard values to predict osteoporotic bone are not yet available. There might be a bias in recording the trabecular peak torque induced by other load components such as bending. The intent of this study was to evaluate if the previous reported peak torque measurement is applicable in a THA setting [13]. Therefore, the reported measurement method has been carried over and adapted accordingly. In this study, the torque measurement correlated significantly with the DXA measurement.

Further studies, preferable with a larger sample size and performed in a clinical setting are required to make surgical treatment recommendations.

The axis of the femoral neck was estimated using a goniometer to represent the conditions during surgery. The retrieved bone cylinders were located next to the region of torque measurement. The bone stock at the proximal femur had been weakened by the retrieval of the bone cylinders.

In order to enable a comparison of the values and to guarantee no cortical contact, the trabecular torque measurement requires an intact femoral head. Therefore, in dysplastic or fractured femoral heads, the torque measurement is not applicable.

## Conclusions

We evaluated an existing intraoperative torque measurement method to assess bone quality and bone strength. We modified the approach to use this method in THA. As there are several approaches used in THA to expose the hip joint, we evaluated the torque in the femoral head as well as femoral neck in order to allow the use in all surgical THA approaches. The results of the trabecular torque measurement showed a significant correlation with the bone mineral density captured by dual energy X-ray absorptiometry (DXA) and with the results of high-sensitivity bone-quality-evaluating methods (monoaxial compressive load test and micro-computed tomography). Hence, the use of this intraoperative torque measurement seems to be a reasonable tool to evaluate bone strength and bone quality for THA. The torque measurement provides sensitive information about the bone strength, which may affect the choice of implant in cases of poor bone stock and osteoporosis, since the surgeon may opt to use a different prosthesis or anchoring technique if poor bone quality is determined. Furthermore, we assume that the disadvantages associated with DXA scans like radiation exposure or errors caused by potential extraosteal sclerosis and interindividual soft-tissue artifacts could be excluded [9,10]. Further studies are required to provide standard values, which would indicate poor bone quality and osteopenia. In addition to that, more research and clinical trials are necessary before this method will turn into clinical use.

## Abbreviations

BMD: bone mineral density
BMI: body mass index
BV: bone volume
DXA: dual energy x-ray absorptiometry
HU: Hounsfield units
μCT: micro-computed tomography
ROI: region of interest
TV: tissue volume
Tb.N: trabecular number
Tb.Th: trabecular thickness
Tb.Sp: trabecular separation
THA: total hip arthroplasty
VOI: volume of interest
